# Effect of Skin Model on* In Vitro* Performance of an Adhesive Dermally Applied Microarray Coated with Zolmitriptan

**DOI:** 10.1155/2018/7459124

**Published:** 2018-06-03

**Authors:** Mahmoud Ameri, Hayley Lewis, Paul Lehman

**Affiliations:** ^1^Zosano Pharma, 34790 Ardentech Court, Fremont, CA 94555, USA; ^2^QPS LLC, Dermal and Transdermal Research Laboratory, 4837 Amber Valley Parkway, Fargo, ND 58104, USA

## Abstract

Franz cell studies, utilizing different human skin and an artificial membrane, evaluating the influence of skin model on permeation of zolmitriptan coated on an array of titanium microprojections, were evaluated. Full thickness and dermatomed* ex vivo* human skin, as well as a synthetic hydrophobic membrane (Strat-M®), were assessed. It was found that the choice of model demonstrated different absorption kinetics for the permeation of zolmitriptan. For the synthetic membrane only 11% of the zolmitriptan coated dose permeated into the receptor media, whilst for the dermatomed skin 85% permeated into the receptor. The permeation of zolmitriptan through full thickness skin had a significantly different absorption profile and time to maximum flux in comparison to the dermatomed skin and synthetic model. On the basis of these results dermatomed skin may be a better estimate of* in vivo* performance of drug-coated metallic microprojections.

## 1. Introduction

The* in vitro*, Franz, human skin finite dose model has proven to be a valuable tool for the study of percutaneous absorption and the determination of the pharmacokinetics of topically applied drugs. The model uses* ex vivo*, human skin mounted in specially designed diffusion cells allowing the skin to be maintained at a temperature and humidity that match typical* in vivo* conditions [1]. A finite dose (for example, 2 mg/cm^2^ – 10 mg/cm^2^ of a semisolid or a transdermal delivery system) of formulation is applied to the outer surface of the skin and drug absorption is measured by monitoring its rate of appearance in the receptor solution bathing the inner surface of the skin. Data defining total absorption, rate of absorption, and skin content can be determined in this model. The method has historic precedent for accurately predicting* in vivo* percutaneous absorption kinetics [2, 3]. The Franz cell has also been applied to assess microneedle mediated drug delivery, utilizing* ex vivo* skin of varying thickness as the diffusional barrier [4–6]. Synthetic membranes such as Silescol® have also been suggested to be a possible alternative to* ex vivo* skin [7, 8]. The perceived advantages of utilizing synthetic membranes is their accessibility, ease of use, and ease of storage, and they may potentially decrease the variability that is associated with utilization of* ex vivo* skin [9]. Though artificial membranes may have some advantages over human skin, unambiguous correlation to human stratum corneum barrier function has yet to be fully explored, particular for finite dose applications [10].

**Figure 1:**
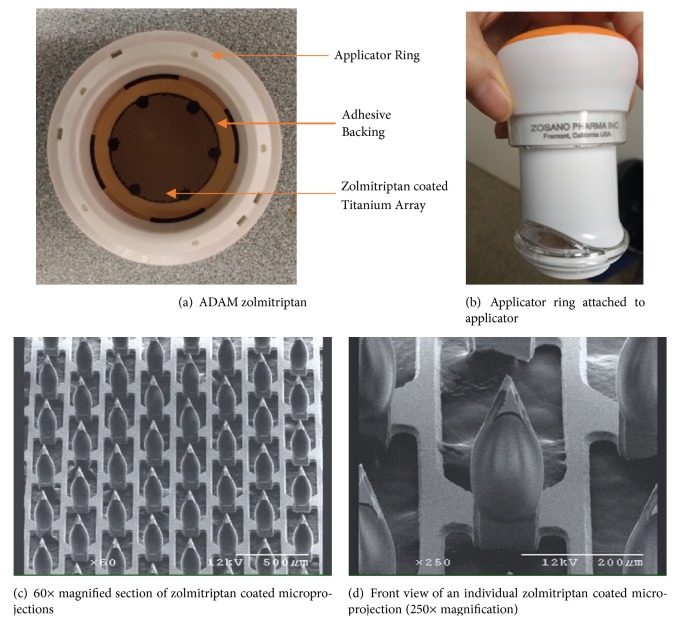
Adhesive Dermally Applied Microarray (ADAM) zolmitriptan. (a) 5 cm^2^ adhesive backing with microprojection array (3 cm^2^) in applicator ring. (b) Applicator ring press fit onto the bottom of the applicator, and applicator. (c) 60× magnification of zolmitriptan coated microprojections (725 microprojections/cm^2^ and length 340 *µ*m), zolmitriptan coated at 1.9 mg/3cm^2^ array. (d) Front view of an individual zolmitriptan coated microprojection (250× magnification).

In the current study, we evaluated Strat-M (synthetic membrane), full thickness, and dermatomed* ex vivo* skin on percutaneous delivery of zolmitriptan from a novel drug-coated microprojections that target the epidermal/dermal layer for rapid and efficient delivery. The Adhesive Dermally Applied Microarray (ADAM) system consists of a titanium microneedle array attached to an adhesive backing seated in a retainer ring, and an applicator (Figure 1(a)). The adhesive backing in the retainer ring is attached to the bottom of the applicator. The applicator (Figure 1(b)) is actuated through spring force, which breaks the adhesive from the retainer ring and applies the patch onto the skin site. The drug-coated microneedles physically break through the stratum corneum and penetrate into the epidermis and dermis, where the dry drug coating is dissolved by the surrounding skin interstitial fluid. The ADAM system has been recently tested in a Phase 2 clinical study with the delivery of zolmitriptan for the treatment of migraines [11].

## 2. Materials and Methods

### 2.1. Study Test Articles

The test system consisted of an ADAM with a 3 cm^2^ titanium array with a nominal dose of 1.90 ± 0.05 mg (0.05 mg denotes the standard deviation of the coated dose) zolmitriptan attached to a 5 cm^2^ adhesive backing. The adhesive backing was seated onto an applicator ring, which had co-molded desiccant. The ADAM zolmitriptan was in a nitrogen purged heat sealed pouch.

### 2.2. Membrane Sources

Dermatomed human skin was obtained from the New York Presbyterian Hospital Skin Bank (NY, NY) and procured from the posterior trunk from 3 donors (2 males, 1 female; 2 Caucasian, 1 African-American; ages of 50 and 51 yrs). This skin was provided to the testing facility as dermatomed, cryopreserved, and sealed in a water impermeable bag with continuous storage at −70°C. All skin was used within its labeled expiration date.

Full thickness human skin was obtained from Science Care (Phoenix, AZ) and procured from the upper outer arm from 1 donor (male, Caucasian, 40 yrs). This skin was provided to the testing facility as excised full thickness and sealed in a water impermeable bag with continuous storage at −20°C.

Synthetic membrane, Strat-M, was purchased from Millipore Sigma (Burlington, MA) as being 47 mm in diameter with a nominal 300 *μ*m thickness.

### 2.3. Receptor Medium

Normal phosphate buffered saline (pH 7.4 ± 0.1) with 0.008% gentamicin sulfate (PBSg) solution was utilized when the diffusion cells were first mounted and for performance of the skin barrier integrity test. Following the barrier integrity test, the reservoir solution was entirely replaced with the 0.1x phosphate buffered saline (pH 7.4 ± 0.1) with 0.008% gentamicin sulfate (0.1x PBSg). The volume of the receptor medium was 25 mL.

### 2.4. Diffusion Cell Preparation

Absorption was measured using the* in vitro*, Franz finite dose technique. Prior to use, skin was thawed in 37°C water and then rinsed in deionized water to remove any adherent blood or other materials from the surface. Skin from each donor was cut into multiple smaller sections large enough to fit on static Franz diffusion cells with a nominal dosing area of 7 cm^2^. The actual thickness of each skin section was measured in triplicate using a Digital Pocket Thickness Gauge. Each skin section was then mounted onto a diffusion cell. The dermal receptor compartment was filled to capacity with PBSg. The epidermal chamber was left unoccluded with exposure to the ambient laboratory environment. The cells were then placed within a rack system and attached to a water circulation system from which the receptor solution was stirred magnetically at approximately 600 RPM, and its temperature was maintained to achieve a skin surface temperature of 32 ± 1°C.

The Strat-M synthetic membrane was mount directly to the diffusion cell without alteration or modification. The cells were then placed within a rack system and attached to a water circulation system, from which the receptor solution was stirred magnetically at approximately 600 RPM, and its temperature was maintained to achieve a skin surface temperature of 32 ± 1°C.

### 2.5. Barrier Integrity Test

To ensure the barrier integrity of each skin section, its desorption of water was measured for transepidermal water loss (TEWL). A Delfin VapoMeter (Surrey, UK) probe was activated and placed onto the skin surface, and the TEWL value was recorded. Skin mounted in diffusion cells in which TEWL was less than 25 g/m^2^/h was considered acceptable. Skin sections that were determined to be unacceptable for dosing may have been used as non-dosed negative sample control cells, if needed. After the barrier integrity test was complete, the receptor solution was replaced with the designated stock receptor solution of a 0.1x PBSg.

### 2.6. Dose Administration and Sample Collection

Prior to administration of the ADAM zolmitriptan to the skin and membrane sections, a predose (0 hour) sample was collected as the entirety of the receptor solution volume was withdrawn with an approximate 5 mL aliquot of the collected sample saved for subsequent analysis. The receptor solution was replaced with the designated stock receptor solution of 0.1x PBSg. The skin was then temporarily removed from the Franz diffusion cell to allow full access to the epidermal surface. Immediately following ADAM zolmitriptan application, the skin-ADAM combination and the donor compartment (chimney) were replaced onto the receptor compartment of the Franz diffusion cell. At the scheduled sampling time points (3, 5, 10, 15, 30, 45, 60, 90, 120, 150, 180, 210, 240, 270, and 300 minutes), the receptor solution was removed in its entirety and refilled with stock receptor solution, and an approximate 5 mL aliquot of the collected sample was saved for subsequent analysis. For sample analysis, a 5 mL aliquot was lyophilized using vacuum centrifugation (Savant™ SpeedVac™) and reconstituted in 0.25 mL of deionized water. After the last receptor sample was collected, the ADAM zolmitriptan was removed for subsequent extraction and analysis. The skin surface wash was performed using two successive refluxing washes of deionized water. Each wash cycle consisted of at least 10 refluxes. The two wash volumes from each donor cell were pooled to generate a single surface wash sample for each diffusion cell. Following the surface wash, the skin was allowed to dry for no less than 10 minutes. Subsequently, the skin was tape-stripped with up to ten sequential tapes (3M Transpore® tape) to remove and collect the stratum corneum. Tape strips were extracted overnight in deionized water on a horizontal mixer (60 rpm) at ambient temperatures. The skin was then dismounted from the cell and separated by manual dissection into epidermis and dermis for subsequent extraction and analysis. Skin sections were extracted overnight in deionized water on a horizontal mixer (~60 rpm) at ambient room temperature.

Strat-M and the ADAM zolmitriptan array were extracted overnight in deionized water on a horizontal mixer (60 rpm) at ambient temperatures.

### 2.7. Dosing

The ADAM zolmitriptan systems were applied to the substrate (cadaver skin or Strat-M membrane) using a hand held reusable applicator (total energy = 0.26 J). The ADAM zolmitriptan was attached to the applicator, and the applicator was pressed on the substrate, releasing the patch and applying it with a predetermined force using a previously described method [12]. Following dosing, the substrate with the adhered ADAM zolmitriptan was immediately mounted onto a Franz diffusion cell.

### 2.8. Sample Analysis

Quantification of zolmitriptan in the collected samples was accomplished using a fully validated HPLC method. Samples were analyzed on a Shimadzu Series LC System. The HPLC/UV method used a solvent system consisting of a mobile phase gradient consisting of (Solvent A) 0.1% ammonium acetate with 0.1% acetic acid in water and (Solvent B) methanol and was run through a Phenomenex Luna C18(2) column (100 x 4.6 mm, 3 *μ*) at a flow rate of 0.5 mL/min for the analysis of zolmitriptan. The column was maintained at 40°C.

### 2.9. Statistical Evaluation

Replicates within skin donors (2 replicates/donor) were averaged and the standard deviation was calculated for each key parameter. Within donor, averages were then collated and the across donor population mean with standard error was calculated. Strat-M was conducted with 6 replicates, which were averaged, and the standard deviation calculated.

## 3. Results

Franz cell experiments are characteristically utilized to assess both* in vitro* bioavailability and bioequivalence of topical semisolid formulations and transdermal systems. One important use is to optimize formulations to enhance percutaneous delivery [13]. Thus, drug permeation through* ex vivo* skin should allow an estimate of* in vivo* percutaneous absorption. Nonetheless, in an* ex vivo* environment the lack of a vascular system means that the distance from the stratum corneum to its interface with the receptor media may contain additional unstirred barriers to drug permeation for some molecules. In an* in vivo* model the distance is 200-400 *μ*m from the stratum corneum to the dermis, where the greatest amount of drug is systemically absorbed via the capillaries [14]. Thus an essential attribute of an* ex vivo* percutaneous absorption experiment is a prudent deliberation of the membrane utilized to model* in vivo* skin conditions [13].

**Figure 2:**
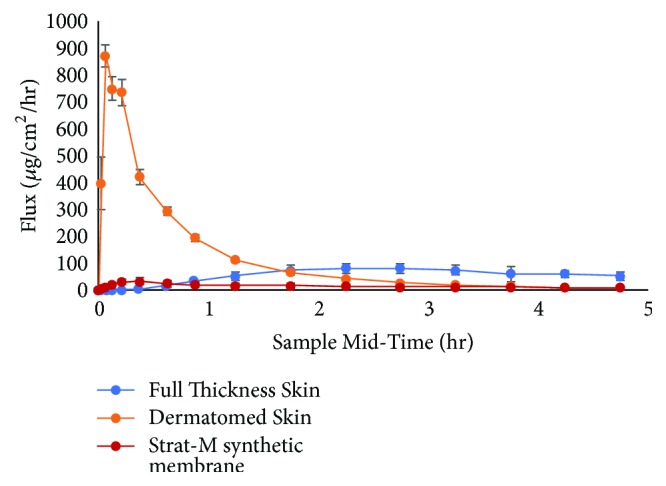
Comparison of mean flux (*μ*g/cm^2^/hr) from full thickness, dermatomed skin, and Strat-M synthetic membrane.

To evaluate the effect of the skin model on microprojection facilitated delivery, full thickness skin (0.70 ± 0.09 mm thickness), dermatomed skin (0.46 ± 0.09 mm), and Strat-M (0.30 ± 0.01 mm thickness) were utilized.  Figure 2 compares the percutaneous absorption of zolmitriptan through full thickness, dermatomed* ex vivo* human skin, and Strat-M synthetic membrane over five hours.

The absorption of zolmitriptan from the two skin sources is markedly different. The time to maximum flux was much slower with the full thickness skin in comparison to the dermatomed skin. The time to maximum flux and the shape of the curve for the full thickness skin substrate are in stark contrast to what has been reported by Kellerman et al. [15] in a Phase 1 clinical study of ADAM zolmitriptan, where the median time to maximum drug plasma concentration was 20 minutes. The difference in the time to peak flux is that with* in vivo* skin, the tips of the microprojections would be very close to the capillary plexus of the dermis allowing for immediate release of drug into the blood stream. Full thickness i*n vitro* skin lacks the blood flow, resulting in the drug having to diffuse through the full dermis to the receptor solution. Utilization of dermatomed skin, where the lower region of the dermis, at the capillary bed, has been removed, is a better* in vitro* skin model as when the drug reaches that area, it finds the receptor solution rather than the capillary blood flow, which, in either case, provides sink conditions. The results indicate that the utilization of dermatomed skin resulted in the greatest extent of intracutaneous delivery in comparison to full thickness skin. That data suggests that the diffusion of zolmitriptan across the full thickness skin demonstrates how the dermis, without capillary flow, will result in a rate limiting step in absorption of microprojection mediated drug delivery [16] attributable to the increased diffusional path length associated with the utilization of full thickness skin [17].

Figure 2 compares the percutaneous absorption of zolmitriptan through Strat-M synthetic membrane with that through dermatomed* ex vivo* human skin over five hours.

Though the Strat-M membrane and the dermatomed skin share a similar permeation curve profile, the peak flux for the Strat-M is significantly lower than that for the dermatomed skin. This phenomenon of very low absorption across synthetic membranes has been reported elsewhere and was attributed to the high elasticity of the synthetic membrane causing the microprojection to retract, such that microprojections do not reside within the created conduits [8]. In addition, adsorption of the zolmitriptan to components of the synthetic membrane,* ca.* 40% of the applied dose recovered in the membrane versus <1% in the epidermal and dermal layers of the dermatomed skin, may also be a contributing factor. Consequently, the utilization of Strat-M membrane does not accurately depict the absorption* in vivo* and may lead to a significant underestimation of the drug release profile.

Table 1 shows the mass balance of 1.9 mg ADAM zolmitriptan that was administered to dermatomed skin and to the Strat-M membrane. In this case, for the skin, the total recovery was 92%, and the total absorbed zolmitriptan through the dermatomed skin to the receptor solution was found to be 85%. Negligible amount of drug was found on the stratum corneum and on the titanium array after administration.

In stark contrast to the skin data presented in Table 1, the amount found in the receptor media for the Strat-M condition was* ca.* 11% of the nominal 1.9 mg zolmitriptan dose. The majority of the dose resided on the titanium array (*ca.* 53%), whilst the remainder was on the surface or within the Strat-M membrane (*ca.* 40%).

## 4. Conclusions 

This study confirmed the influence of choice of* in vitro* experimental conditions on the rate and extent of permeation of ADAM zolmitriptan. The results of the present study suggest that synthetic membranes such as Strat-M should be employed with caution when evaluating drug release from coated microprojections that are designed to deposit their drug locally* via* dissolution. Dermatomed skin may be not only a more representative measure of* in vivo *performance for drug-coated metallic microprojections, but also a more representative approach for most* in vitro* absorption studies.

## Figures and Tables

**Table 1:** Mass balance results, as percent of applied dose (%) of zolmitriptan into and through *ex vivo* human dermatomed skin and Strat-M membrane over 5 hours from a single application. Mean ± SE for skin (n=3 donors with 3 replicates/donor) and mean ± SD (6 replicates) for the membrane.

**Parameter**	**Mean Zolmitriptan (%)** **Dermatomed Skin**	**Mean Zolmitriptan (%)** **Strat-M**
Receptor	85.46 ± 1.36	10.55 ± 6.15
Dermis	0.50 ± 0.04	- - -
Epidermis	0.43 ± 0.16	- - -
Stratum Corneum	0.13 ± 0.04	- - -
Surface Wash	2.57 ± 1.09	- - -
Strat-M Extraction	- - - -	39.76 ± 19.75
Ti Array	3.27 ± 0.36	52.79 ± 29.28
Total Recovery	92.35 ± 2.48	103.1 ± 9.2
